# Complete chloroplast genomes and comparative analysis of *Ligustrum* species

**DOI:** 10.1038/s41598-022-26884-7

**Published:** 2023-01-05

**Authors:** Lianxiang Long, Yongtan Li, Shijie Wang, Zhenlin Liu, Jinmao Wang, Minsheng Yang

**Affiliations:** 1 Institute of Forest Biotechnology, Forestry College, Agricultural University of Hebei, Baoding, 071000 China; 2 Hebei Key Laboratory for Tree Genetic Resources and Forest Protection, Baoding, 071000 China; 3 College of Horticulture Science and Technology, Hebei Normal University of Science & Technology, Changli, 066600 Hebei China

**Keywords:** Genomics, Genetic markers, Phylogenetics

## Abstract

In this study, we assembled and annotated the chloroplast (cp) genomes of four *Ligustrum* species, *L. sinense*, *L. obtusifolium*, *L. vicaryi*, and *L. ovalifolium* ‘Aureum’. Including six other published *Ligustrum* species, we compared various characteristics such as gene structure, sequence alignment, codon preference, and nucleic acid diversity, and performed positive-selection genes screening and phylogenetic analysis. The results showed that the cp genome of *Ligustrum* was 162,185–166,800 bp in length, with a circular tetrad structure, including a large single-copy region (86,885–90,106 bp), a small single-copy region (11,446–11,499 bp), and a pair of IRa and IRb sequences with the same coding but in opposite directions (31,608–32,624 bp). This structure is similar to the cp genomes of most angiosperms. We found 132–137 genes in the cp genome of *Ligustrum*, including 89–90 protein-coding genes, 35–39 tRNAs, and 8 rRNAs. The GC content was 37.93–38.06% and varied among regions, with the IR region having the highest content. The single-nucleotide (A/T)n was dominant in simple-sequence repeats of the *Ligustrum* cp genome, with an obvious A/T preference. Six hotspot regions were identified from multiple sequence alignment of *Ligustrum*; the *ycf1* gene region and the *clpP1* exon region can be used as potential DNA barcodes for the identification and phylogeny of the genus *Ligustrum*. Branch-site model and Bayes empirical Bayes (BEB) analysis showed that four protein-coding genes (*accD*, *clpP*, *ycf1*, and *ycf2*) were positively selected, and BEB analysis showed that *accD* and *rpl20* had positively selected sites. A phylogenetic tree of Oleaceae species was constructed based on the whole cp genomes, and the results were consistent with the traditional taxonomic results. The phylogenetic results showed that genus *Ligustrum* is most closely related to genus *Syringa*. Our study provides important genetic information to support further investigations of the phylogenetic development and adaptive evolution of *Ligustrum* species.

## Introduction

There are approximately 50 *Ligustrum* (Oleaceae) species worldwide, mainly distributed in warm regions of Asia and extending northwest to Europe and south to New Guinea and Australia via Malaysia^1^. Among these, approximately 38 species are distributed in China, mainly in the south and southwest. This genus comprises evergreen, semi-evergreen, or deciduous trees and shrubs with opposite, simple leaves with papery or leathery blades^2^. *Ligustrum* species thrive in light and are slightly shade tolerant and relatively cold tolerant; their dense, pruning-tolerant branches have been used extensively as decorative hedging material with high ornamental value. *Ligustrum* species also have medicinal value; e.g., *Ligustrum lucidum* leaves can be distilled to extract wintergreen oil, which is used as an additive in foods and toothpaste. Its dried fruits are also used as the traditional Chinese medicine lucidum, which is cool and bittersweet, and brightens the eyes and hair and nourishes the liver and kidneys^3,4^. *Ligustrum* species also effectively adsorb atmospheric pollutants such as SO_2_ and NO_2_ and exhibit strong stress resistance, playing a positive role in purifying the air and improving regional ecological quality^5^. However, research on *Ligustrum* species has mainly focused on morphology, physiology, population characteristics, and pharmacological activity, with few studies investigating the molecular basis for germplasm identification, genetic breeding, resource conservation, and phylogenetics, which can affect the conservation and exploitation of *Ligustrum* species. Therefore, to elucidate the taxonomic relationships and positions of *Ligustrum* species within family Oleaceae, and to more effectively conserve and use *Ligustrum* species, further in-depth studies are required.

With the recent development of high-throughput sequencing technology, cp genome sequencing technology has gradually improved. Chloroplasts are organelles in green plants involved in photosynthesis, as well as vitamin, starch, protein, and pigment synthesis. The cp genome is inherited autonomously and has played a critical important role in plant evolutionary history. In most angiosperms, the cp genome is inherited maternally, with only a few species exhibiting biparental or paternal inheritance^6^. The structure and sequence of the cp genome are relatively conserved, and most of the genome structure consists of a double-stranded loop that includes two inverted repeat regions (IRa/IRb), one large single-copy (LSC) region and one small single-copy (SSC) region, generally ranging in size from 120 to 180 kb^7^. Although the cp genome is relatively conserved in terms of gene composition and structure compared with the nuclear and mitochondrial genomes, recent studies have identified many genetic mutations in the cp genome, such as the loss of genes or intron fragments^8,9^, variation in reverse repeat region length or insertion/deletion of partial fragments^10^, expansion or deletion of entire reverse repeat regions^10^, and gene rearrangement^11,12^. Because the cp genome is the smallest genome in plant cells, with easily accessible full sequences, conserved genome structure, and stable gene composition, it has become an ideal model for evolutionary and comparative genomic studies^13,14^, providing a basis for investigating phylogenetic positions and genetic–developmental relationships among plant taxa. The cp genomes have been increasingly reported, and these complete cp genome sequences provide better data to distinguish marginal groups, especially below the species level. The cp genome contains a large amount of genetic information, which contributes to advances in comparative genomics and phylogenetics. In particular, comparative analysis based on cp genome data can explain the evolution and phylogenetic relationships of species more comprehensively than one or a few DNA fragments^15^. However, the cp genomes of many families remain to be published; therefore, it is necessary to further investigate cp genome information to resolve phylogenetic relationships among more plant species.

In this study, to obtain further genetic information about genus *Ligustrum*, we spliced, assembled, and annotated the chloroplasts of *L. sinense*, *L. obtusifolium*, *L. vicaryi*, and *L. ovalifolium* ‘Aureum’, and investigated their characteristics in comparison with those of six other published *Ligustrum* species. The objectives of this study were to provide whole-chloroplast genome data for four *Ligustrum* species, compare the genome structure and sequence variation of their cp genomes, investigate simple and large repetitive sequences and hotspot regions as candidate sequences for *Ligustrum* species identification and phylogenetic studies, identify positively selected genes as potential genes for adaptive evolution in the genus, and use cp genome sequences of 37 Oleaceae species to construct a phylogenetic tree to clarify their phylogenetic relationships.

## Results

### Chloroplast genome structures of *Ligustrum* species

**Figure 1:**
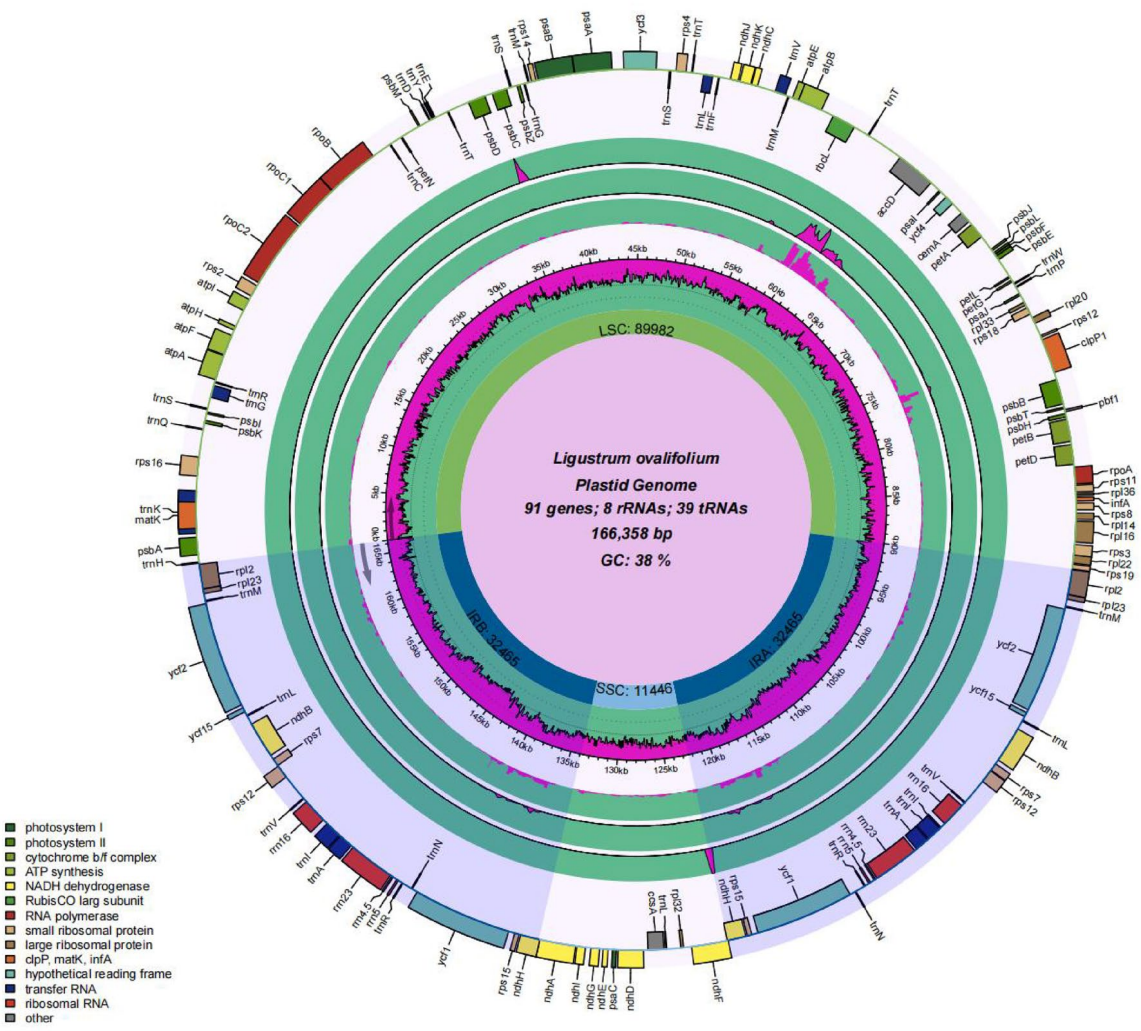
Chloroplast genome maps of four *Ligustrum* species. The species name and specific information regarding the genome (length, GC content, and the number of genes) are depicted in the center of the plot. From outside to inside, the outermost: *L.ovalifolium* 'Aureum', and then followed by *L. sinense, L. obtusifolium* and *L. vicaryi* compared with *L.ovalifolium* 'Aureum', respectively.

The cp genomes of all four *Ligustrum* species were covalently closed double-stranded circular molecules, including a pair of sequences with the same coding but in the opposite orientation (IRa and IRb), one LSC region, and one SSC region. No deletions of large segments or regional bases were detected. The genome length ranged from 162,272 to 166,358 bp (Fig. 1). There were heteroplasmy. When each species is compared with *L. sinense*, different SNPs will be obtained. The cp genome length of *L. obtusifolium* and *L. sinense* was 815 bp different, and there were 291 SNPs in total. The cp genome length of *L. vercaryi* and *L. sinense* was 3996 bp different, and there were 274 SNPs in total. The cp genome length of *L. ovalifolium* ‘Aureum’ and *L. sinense* differed by 4086 bp, with a total of 284 SNPs (Supplemental file-SNP). Although heteroplasmy exists, but there is little difference in the type and number of cp genes (Table 1). The cp genomes of the four *Ligustrum* species are relatively conserved.

**Table 1:** The basic characteristics of the chloroplast genomes of ten *Ligustrum* species.

	*L. sinense*	*L. obtusifolium*	*L. vicaryi*	*L. ovalifolium 'Aureum'*	*L. gracile*	*L. quihoui*	*L. japonicum*	*L. lucidum*	*L. ovalifolium*	*L. vulgare*
GenBank accession number	This study	This study	This study	This study	NC_042425.1	NC_057246.1	NC_042454.1	NC_056243.1	NC_056242.1	NC_042274.1
Total length (bp)	162,272	163,087	166,268	166,358	163,110	163,575	163,562	162,498	166,800	162,185
Total GC (%)	37.95	37.93	37.95	37.95	38.06	37.94	37.93	38.06	37.98	37.94
LSC Length (bp)/(%)	86,885	87,948	89,992	89,982	88,239	88,072	88,214	87,540	90,106	87,497
GC (%)	36.28	36.17	36.29	36.27	36.33	36.22	36.21	36.33	36.29	36.25
SSC Length (bp)/(%)	11,461	11,481	11,470	11,446	11,499	11,477	11,486	11,486	11,446	11,485
GC con10t (%)	32.68	32.76	32.78	32.79	32.72	32.81	32.68	32.78	32.79	32.71
IR Length (bp)/(%)	31,963	31,829	32,403	32,465	31,686	32,013	31,931	31,736	32,624	31,608
GC con10t (%)	41.16	41.28	41.19	41.17	41.43	41.23	41.24	41.40	41.21	41.23
Coding region length (bp)	85,359	85,179	84,903	87,273	86,478	85,023	86,865	85,842	89,070	86,523
Coding region GC (%)	38.04	38	38.04	38.05	38.19	38.04	38.09	38.16	38.22	38.01
Noncoding region length (bp)	76,913	77,908	81,365	79,085	76,632	78,552	76,697	76,656	77,730	75,662
Noncoding region GC (%)	37.85	37.85	37.86	37.84	37.91	37.83	37.75	37.95	37.7	37.86
Protein-coding gene num	89	90	89	90	90	90	90	89	90	90
Protein-coding region GC (%)	38.04	38	38.04	38.05	38.19	38.04	38.09	38.16	38.22	38.01
rRNA GC (%)	55.37	55.34	55.37	55.37	55.26	55.32	55.32	55.22	55.37	55.28
tRNA GC (%)	52.97	52.98	52.87	52.97	52.88	52.88	53.03	52.88	52.81	52.96
Total tRNA	37	37	38	39	35	35	35	35	36	35
Total rRNA	8	8	8	8	8	8	8	8	8	8
Total gene num	134	135	135	137	133	133	133	132	134	133

**Figure 2:**
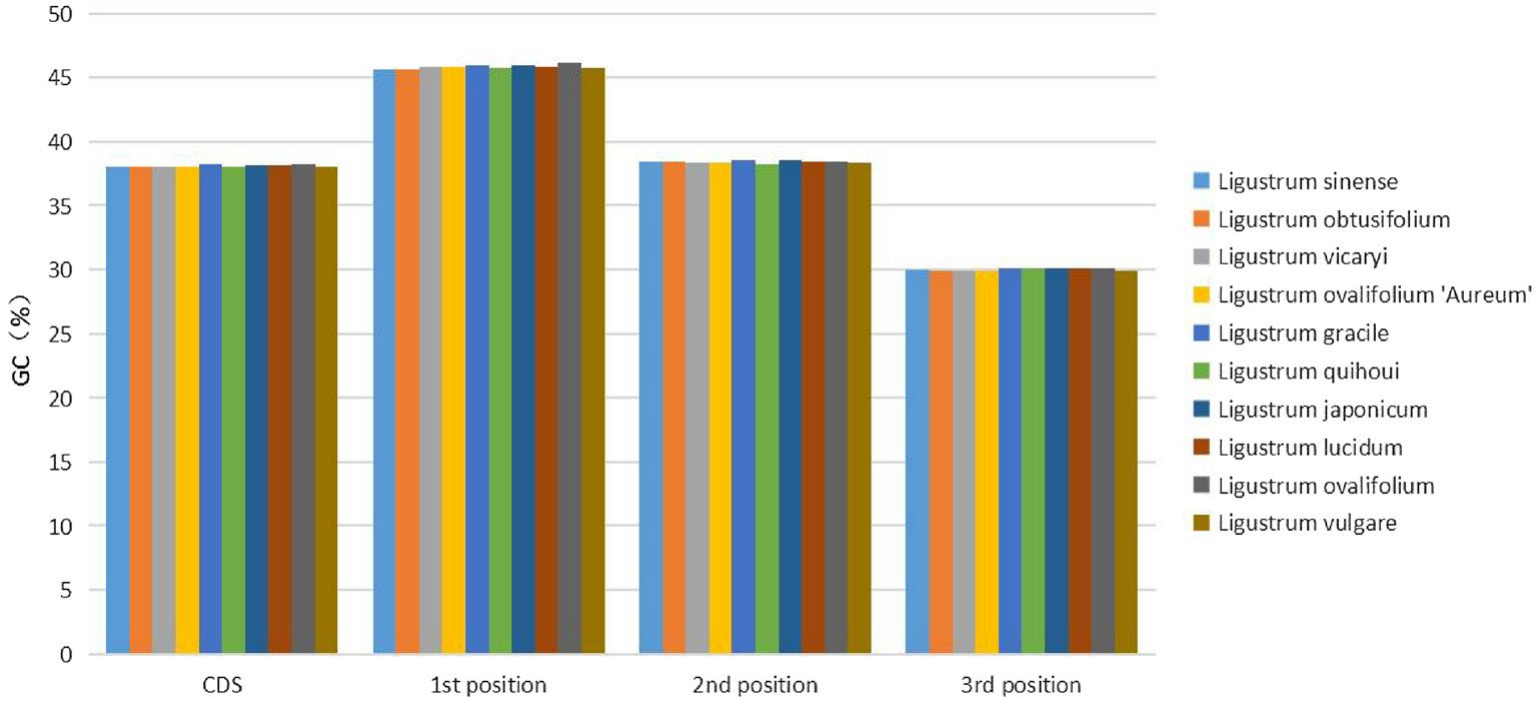
The GC (%) composition in different positions of coding sequence (CDS) region of ten *Ligustrum* species.

Next, the basic characteristics of the cp genomes of ten *Ligustrum* plants were evaluated. The total length of *Ligustrum* cp genomes ranged from 162,185 bp (*L. vulgare*) to 166,800 bp (*L. ovalifolium*). The length of the LSC region ranged from 86,885 bp (*L. sinense*) to 90,106 bp (*L. ovalifolium*); the SSC region length ranged from 11,446 bp (*L. ovalifolium*, *L. ovalifolium* ‘Aureum’) to 11,499 bp (*L. gracile*), the IR region length ranged from 31,608 bp (*L. vulgare*) to 32,624 bp (*L. ovalifolium*), the coding region length ranged from 84,903 bp (*L. vicaryi*) to 89,070 bp (*L. ovalifolium*), and the non-coding region length ranged from 75,662 bp (*L. vulgare*) to 81,365 bp (*L. vicaryi*) (Table 1). A total of 132–137 cp genes were detected, comprising 89–90 protein-coding genes, 35–39 tRNA genes, and 8 rRNA genes. GC content differed among positions within the cp genomes, and also different among genes coding different functions, with generally higher GC content in the gene-coding region (38.00–38.22%) than in the non-coding region (37.70–37.91%); GC content was highest in the IR region (41.16–41.40%), followed by the LSC region (36.17–36.33%) and SSC region (32.68–32.81%). The rRNA GC content of the entire coding region was 55.22–55.37%; the total GC content (37.93–38.06%) was lower than that in the IR region but higher than those in the SSC and LSC regions. Among protein-coding sequences, GC content was higher in the first than in the second and third (Fig. 2).

Duplicate genes were counted only once; thus, a total of 114 genes were annotated in the cp genomes of ten *Ligustrum* species, including 82 protein-coding genes, 4 rRNA genes, and 28 tRNA genes (Table 2). Introns play an important role in gene expression regulation. A total of 22 genes in the cp genomes of ten *Ligustrum* species contained introns, among which the genes *ndhA*, *ndhB*, *petB*, *petD*, *atpF*, *rpl2*, *rpl16*, *rps12*, *rps16*, *rpoC1*, *accD*, *trnA-UGC*, *trnG-GCC*, *trnG-UCC*, *trnI-GAU*, *trnL-CAA*, *trnL-UAA*, *trnL-UAG*, *trnV-GAC*, and *trnV-UAC* each contained one intron, and *ycf3* and *clpP* each contained two introns. Only the *accD* gene of *L. obtusifolium* and *L. vicaryi*, contained one intron, whereas the *accD* gene of all other *Ligustrum* species had no introns; similarly, the *trnV* gene of *L. sinense*, *L. obtusifolium*, *L. vicaryi*, and *L. ovalifolium* ‘Aureum’ contained one intron, and the *trnV* gene of all other *Ligustrum* species had no introns. Gene intron loss occurs during the evolution of *Ligustrum* species (Supplementary Table 1).

**Table 2:** List of genes annotated in the chloroplast genomes of ten *Ligustrum* species in this study.

Category of genes	Group of genes	Name of genes	Amount
Photosynthesis	Subunits_of_photosystem_I	*psaA, psaB, psaC, psaI, psaJ*	5
	Subunits_of_photosystem_II	*pbf1, psbA, psbB, psbC, psbD, psbE, psbF, psbG, psbH, psbI, psbJ, psbK, psbL, psbM, psbN, psbT, psbZ*	17
	Subunits_of_NADH_dehydrogenase	ndhA*, ndhB *(× *2), ndhC, ndhD, ndhE, ndhF, ndhG, ndhH, ndhI, ndhJ, ndhK*	11
	Subunits_of_cytochrome_b/f_complex	petA, petB*, petD*, petG, petL, petN	6
	Subunits_of_ATP_synthase	atpA, atpB, atpE, atpF*, atpH, atpI	6
	Large_subunit_of_Rubisco	*rbcL*	1
Self-replication	Large_subunits_of_ribosome	rpl14, rpl16*, rpl2* (× *2), rpl20, rpl22, rpl23 (*× *2), rpl32, rpl33, rpl36*	9
	Small_subunits_of_ribosome	rps11, rps12* (× *2), rps14, rps15(*× 2), rps16*, rps18, rps19, rps2, rps3, rps4, rps7(× *2), rps8*	12
	DNA-dependent_RNA_polymerase	rpoA, rpoB, rpoC1*, rpoC2	4
	Ribosomal_RNAs	*rrn16, rrn23, rrn4.5, rrn5*	4
	Transfer_RNAs	trnA-UGC*(× 2), trnC-GCA, trnD-GUC, trnE-UUC, trnF-GAA, trnG-GCC*, trnG-UCC*, trnH-GUG, trnI-GAU*(× 3), trnK-UUU, trnL-CAA* (× 2), trnL-UAA*, trnL-UAG*, trnM-CAU(× *4), trnN-GUU(*× *2), trnP-UGG, trnQ-UUG, trnR-ACG(*× *2), trnR-UCU, trnS-GCU, trnS-GGA, trnS-UGA, trnT-GGU(*× 2), trnT-UGU, trnV-GAC*(× 2), trnV-UAC*, trnW-CCA, trnY-GUA	28
Other genes	Maturase	*matK*	1
	Protease	clpP**	1
	Envelope_membrane_protein	*cemA*	1
	Acetyl-CoA_carboxylase	accD*	1
	C-type_cytochrome_synthesis_gene	*ccsA*	1
	Translation_initiation_factor	*infA*	1
Genes of unknown	Proteins_of_unknown_function	*ycf1(*× *2), ycf15 (*× *2), ycf2(*× 2), ycf3**, ycf4	5

*Gene contains one intron; **Gene contains two introns; (× 2) indicates the number of the repeat unit is 2.

### Codon usage indices

**Figure 3:**
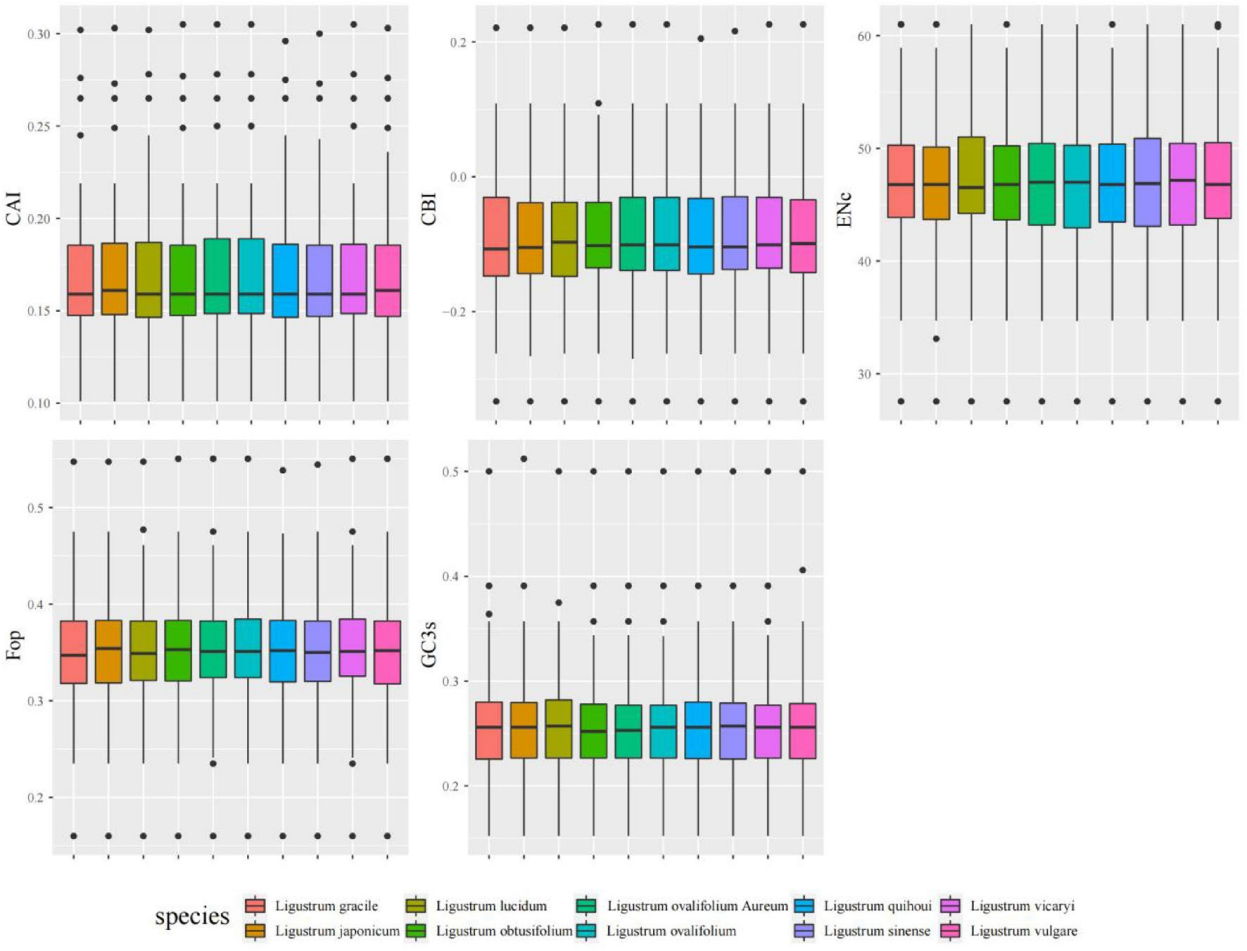
The comparative analysis of codon usage bias in 10 *Ligustrum* species, including Codon adaptation index (CAI), Codon bias index (CBI), Frequency of optimal codons index (Fop), Effective number of codons (ENc), GC of synonymous codons in third position (GC3s).

Investigation of the codon usage preferences of *Ligustrum* species showed that codon adaptation index (CAI), codon bias index (CBI), frequency of optimal codons (FOP), and GC content at the third codon position (GC3) values were similar among the ten *Ligustrum* species, whereas the effective number of codons (ENc) was slightly higher in *L. lucidum* than in other species (Fig. 3). The ENc values of all *Ligustrum* cp protein-coding genes in this study were > 40; based on ENc values between 20 (complete preference) and 61 (no preference)^16^, the overall preference for codon use among *Ligustrum* cp protein-coding genes was weak.

### IR contraction and expansion

**Figure 4:**
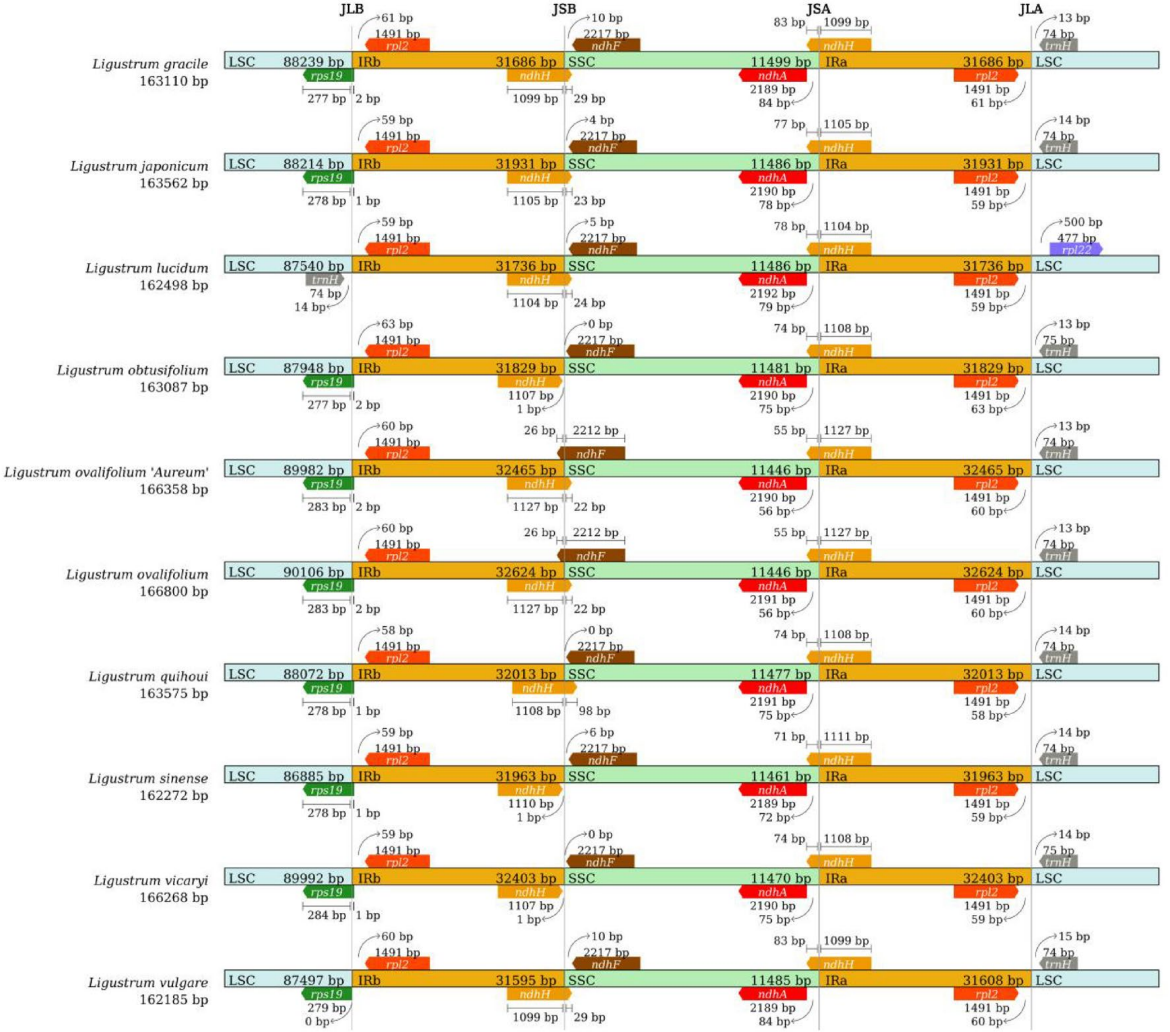
Comparison of the borders of large single-copy (LSC), small single-copy (SSC), and inverted repeat (IR) regions among ten *Ligustrum* chloroplast genome.

The cp genome is a ring structure consisting of the LSC, SSC, IRa, and IRb regions, with four boundaries: LSC–IRb, IRb–SSC, SSC–IRa, and IRa–LSC. Expansion and contraction of the IR region of the cp genome is an important event in plant evolutionary history and causes changes in the size and gene content of the cp genome. In this study, we compared the LSC/IRb/SSC/IRa boundaries of cp genomes from ten *Ligustrum* species (Fig. 4). The genotypes of the IR–LSC and IR–SSC boundaries were essentially the same, with relatively conserved IR lengths among all ten species (31,608–32,624 bp) and no significant amplification or contraction events. The IR–SC boundary differed among the cp genomes of the ten *Ligustrum* species; seven protein-coding genes (*rps19*, *rpl2*, *ndhH*, *ndhF*, *ndhA*, *rpl22*, and *trnH*) were present at the LSC–IR and SSC–IR boundaries. The LSC–IRb boundary of *L. lucidum* was located between *trnH* and *rpl2*, with *trnH* located 14 bp to the left and *rpl2* located 59 bp to the right. In all other species, the LSC–IRb boundary was located between *rps19* and *rpl2*; in the other species, the LSC–IRb boundary extended into *rps19* with a 1–2 bp length variation, except for that of *L. vulgare*, which was immediately adjacent to *rps19*. In *L. obtusifolium*, *L. sinense*, and *L. vicaryi*, *ndhH* was 1 bp to the left of the IRb–SSC boundary; in the other species, the IRb–SSC boundary extended into *ndhH*, with a length variation of 22–98 bp. The IRb–SSC boundary extended into *ndhF* by 26 bp in *L. ovalifolium* ‘Aureum’ and *L. ovalifolium*, was immediately adjacent to *ndhF* in *L. obtusifolium* and *L. quihoui*, and was located 4–10 bp to the right of *ndhF* in the other *Ligustrum* species. The SSC–IRa boundary of all *Ligustrum* species extended into *ndhH*, with a length variation of 74–83 bp; the *ndhA* gene was located 56–84 bp to the left of this boundary. The IRa–LSC boundary of *L. lucidum* was between *rpl2* and *trnH*, with *rpl2* located at a distance of 59 bp; *rpl22* was located 500 bp to the right of the IRa–LSC boundary. In the other *Ligustrum* species, the IRa–LSC boundary was between *rpl*2 and *trnH*; *rpl2* was located 58–63 bp to the left of the IRa–LSC boundary and *trnH* was located 13–15 bp to the right of the IRa–SSC.

### Repeat sequence analysis and simple sequence repeats (SSRs)

**Figure 5:**
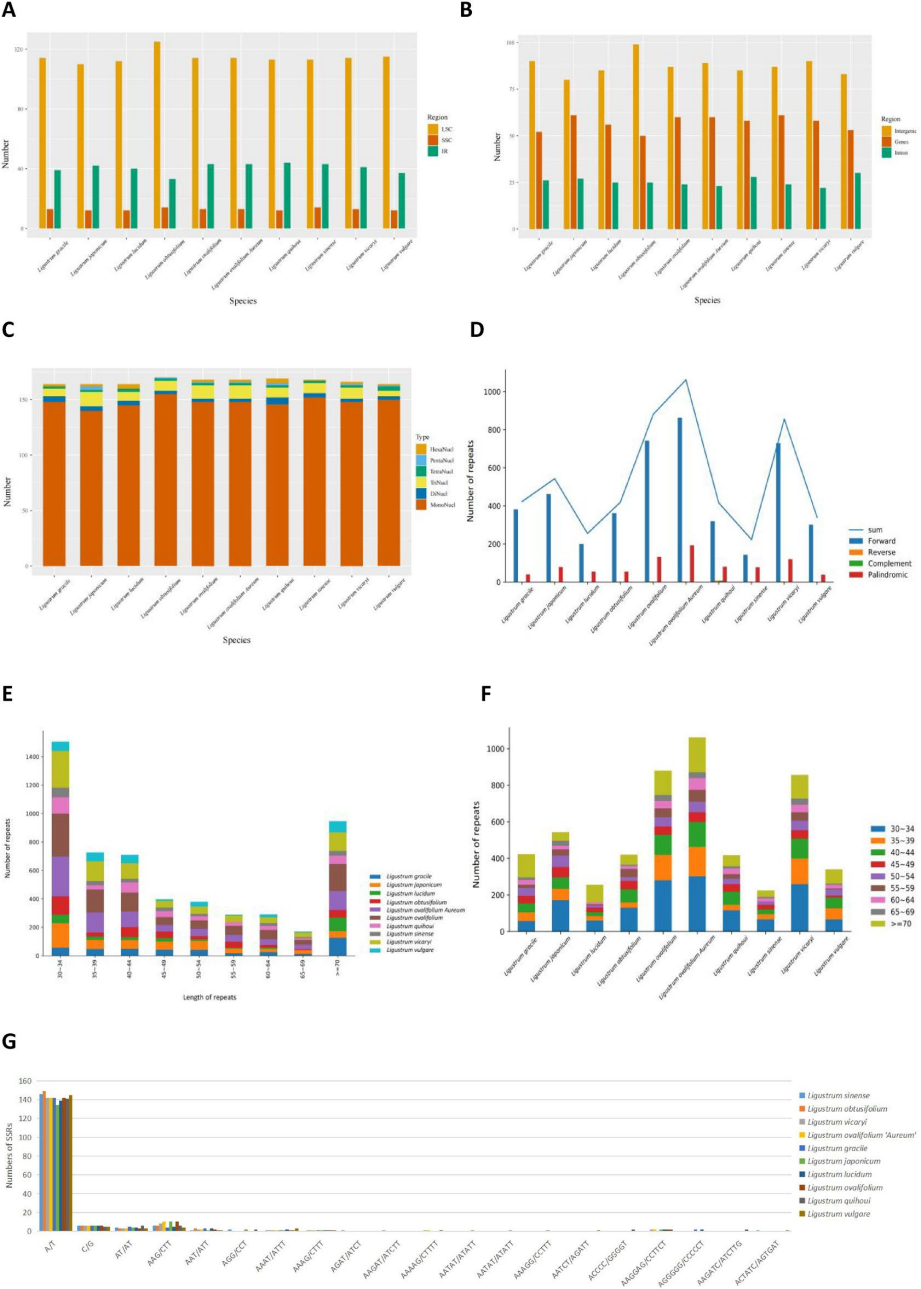
(**A**) Frequency of SSRs in the LSC, IR, SSC region; (**B**) Frequency of SSRs in the intergenic regions, protein-coding genes, and introns; (**C**) Type of SSRs; (**D**) Number of Forward repeat, Reverse repeat, Complement repeat, Palindromic repeat; (**E**) Length distribution of long repeats; (**F**) Length repeat species; (**G**) Analysis of simple-sequence repeats (SSRs) in the chloroplast genomes of ten *Ligustrum* species.

Because SSRs have high polymorphism rates at the species level, they have become an important source of molecular markers, and have been extensively investigated in phylogenetic and population genetics studies. In this study, SSRs were mainly distributed in the LSC and SSC regions of the cp genome (Fig. 5A), which are also major cp distribution regions, with few SSRs in the two IR regions. According to SSR location analysis, most were distributed in the non-coding regions of the genome, i.e., the intergenic and intronic regions (Fig. 5B). A total of 164 (*L. gracile*, *L. lucidum*, *L. japonicum*, and *L. vulgare*) to 170 (*L. obtusifolium*) SSRs were detected in the cp genomes of *Ligustrum* species, which had the largest number of single nucleotides (140–155), dinucleotides (3–6), trinucleotides (5–13), tetranucleotides (2–4), pentanucleotides (1–3), and hexanucleotides (1–4) (Fig. 5C). Single nucleotide repeats may play a more important role in gene variation than other types of SSRs. These SSRs were dominated by single nucleotide (A/T)n (Fig. 5G), suggesting that the base composition of SSRs is biased toward A/T bases.

Long repetitive sequences (≥ 30 bp) may promote cp genome rearrangement and increase the function of species genetic diversity. A total of 223 (*L. sinense*) to 1,062 (*L. ovalifolium*) long repeat sequences were predicted in the *Ligustrum* cp genomes, including 142–862 forward repeats, 1–8 reverse repeats, 1–8 complementary repeats, and 40–194 palindromic repeats (Fig. 5D). The largest number of long repeats was found to have a length of 30–34 bp, and the smallest had a length of 65–69 bp (Fig. 5E). Among these, *L. ovalifolium* ‘Aureum’ had the highest number of long repeat sequences (Fig. 5F). We also detected 44 (*L. vulgare*) to 88 (*L. ovalifolium*) tandem repeats.

### Comparative genomic divergence and hotspot regions

**Figure 6:**
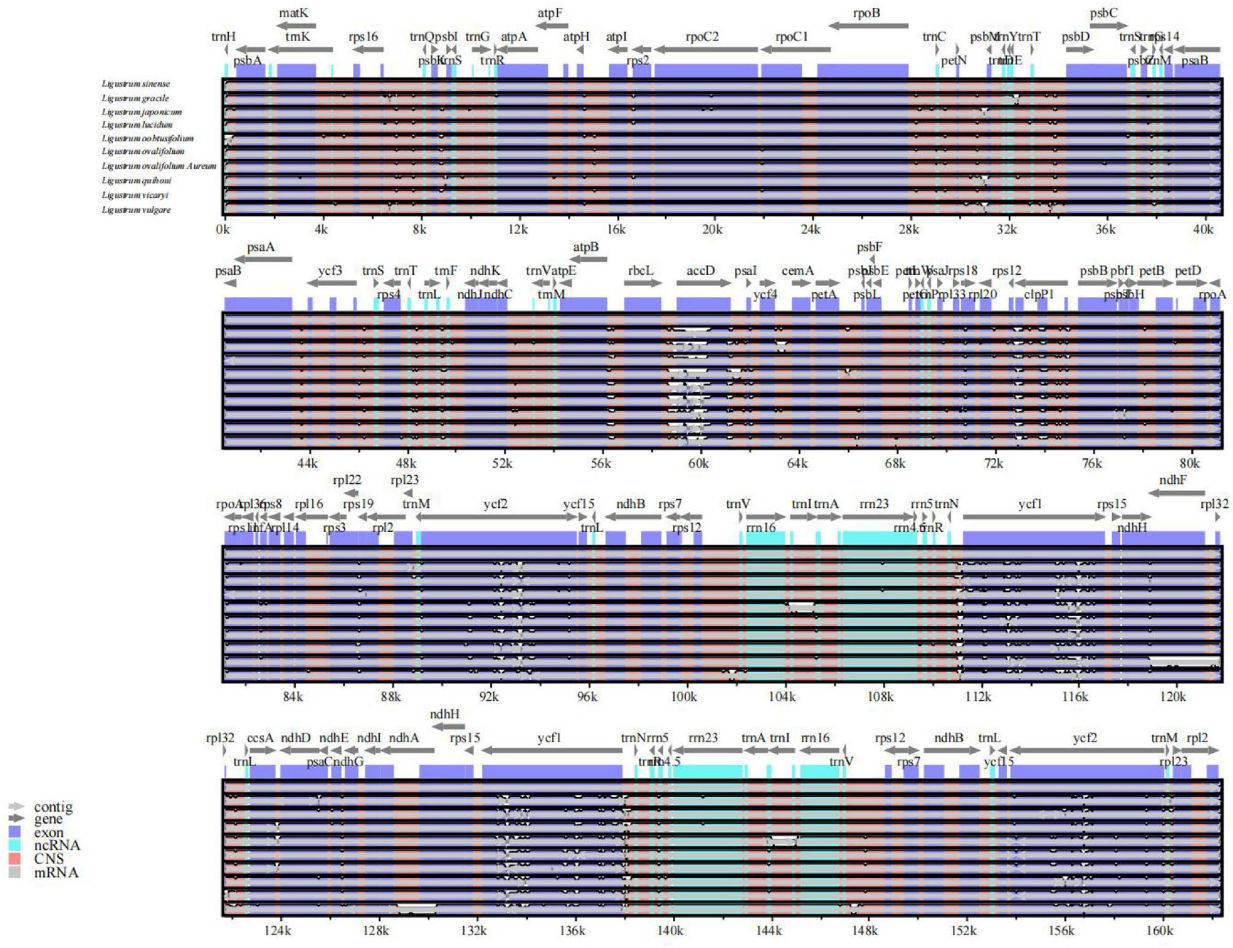
Comparison of ten *Ligustrum* chloroplast genomes using mVISTA, with the *L.sinense* genome as the reference. The y-axis represents the percent identity within 50–100%. Gray arrows indicate the direction of gene transcription. Blue blocks indicate conserved genes, while red blocks indicate conserved noncoding sequences (CNS).

To determine the sequence differences among the ten *Ligustrum* cp genomes, we used *L. sinense* as a reference genome and compared them using the mVISTA software. *Ligustrum* cp whole-genome sequences encoded gene classes, numbers, and alignments that were highly consistent among species. Variation among sequences occurred mainly in non-coding intergenic regions, and coding regions were generally more conserved (Fig. 6).

**Figure 7:**
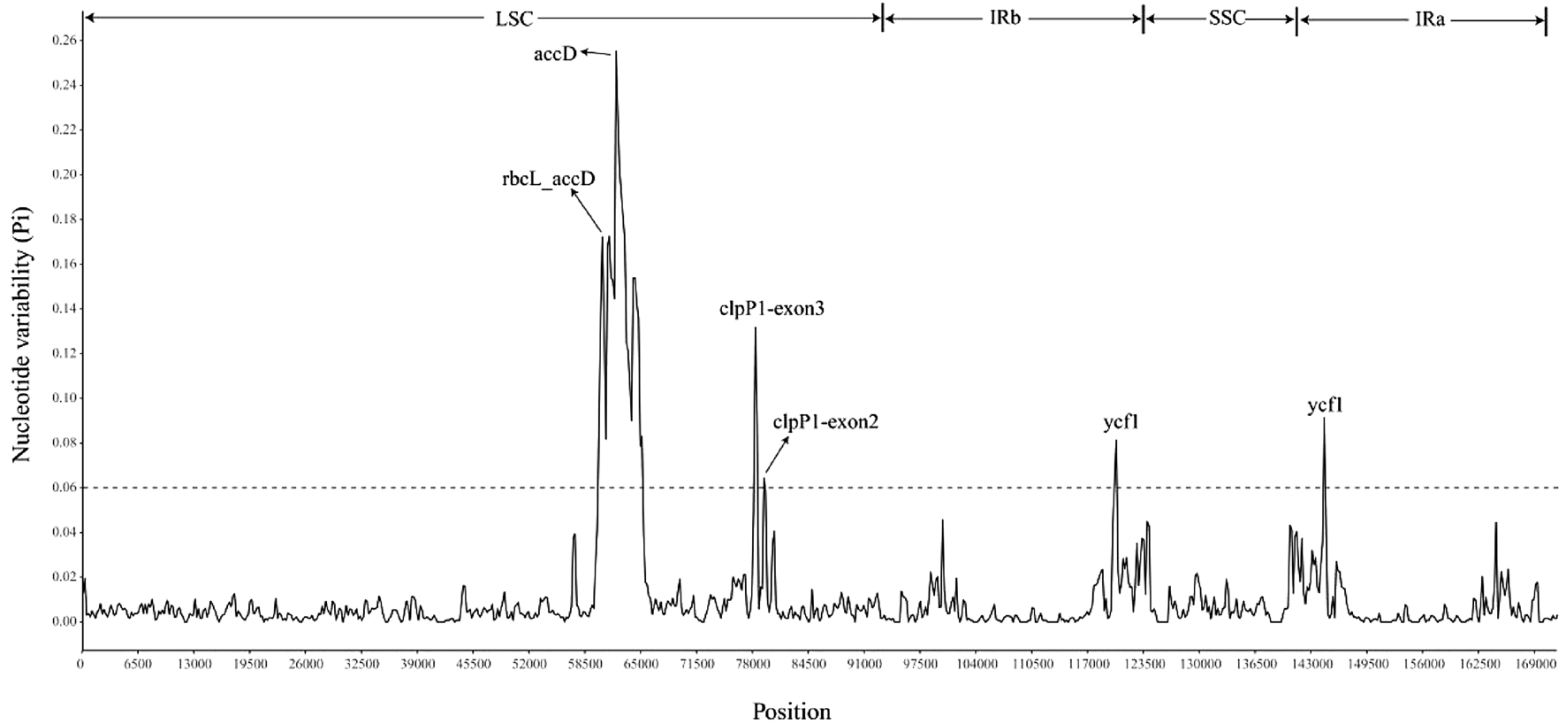
Nucleotide diversity of the ten *Ligustrum* species chloroplast genomes.

Next, we calculated the nucleotide diversity (Pi) of the ten *Ligustrum* species. The high-variation regions of the *Ligustrum* cp genomes were mainly concentrated in the LSC and IR regions. Six regions, i.e., one intergenic region (*rbcL_accD*) and five genic regions (*accD*, *clpP1-exon3*, *clpP1-exon2*, *ycf1*, and *ycf1*), were considered as hotspot regions (Pi > 0.06), among which gene region *accD* had the highest nucleotide diversity (0.2552083), followed by the intergenic region *rbcL_accD* (0.172619) (Fig. 7, Table 3). Four of these hotspot regions were located in the LSC region and two in the IR region. Further analysis of the six hotspot regions showed that *rbcL_accD* intergene region included a large number of insertion and deletion events. There were large fragment deletion and intron loss in *accD* gene, resulting in large sequence difference and difficult sequence alignment. Therefore, it is not recommended as a candidate DNA barcode for the *Ligustrum*. However, the *ycf1* gene region and the *clpP1* exon region not only have high sequence variability, but also are coding region sequences, which can be accurately corrected by triplet codons. Therefore, the *ycf1* gene region and the *clpP1* exon region can be used as potential DNA barcodes for the identification and phylogeny of the *Ligustrum*.

**Table 3:** Highly variable sequences (Pi > 0.06) of ten *Ligustrum* species.

High variable marker	Length	Variable sites	Parsimony informative sites	Nucleotide diversity
*rbcL_accD*	420	192	165	0.172619
*accD*	1016	330	299	0.2552083
*clpP1-exon3*	358	129	104	0.1316551
*clpP1-exon2*	300	68	55	0.0642884
*ycf1*	102	33	31	0.0811966
*ycf1*	91	31	28	0.0914083

### Pairwise comparison of species Ka/Ks ratios and positive selection analyses

**Figure 8:**
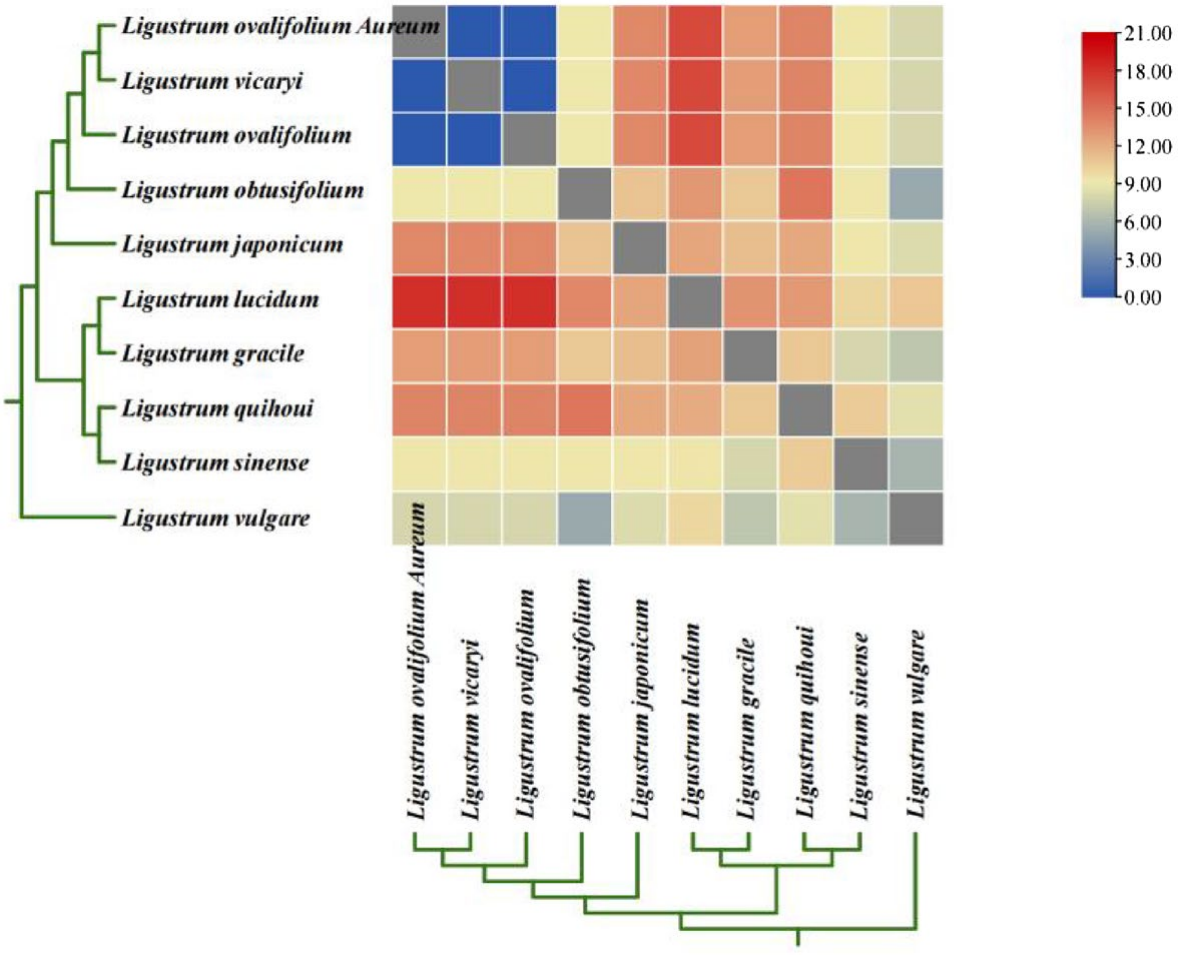
Pairwise Ka/Ks ratios ten *Ligustrum* species. This heatmap shows pairwise Ka/Ks ratios between every sequence in the multigene nucleotide alignment.

The Ka/Ks ratios of *Ligustrum* species were calculated to provide information on the selection pressure acting on individual sequences. Of the ten *Ligustrum* species, *L. lucidum, L. gracile*, and *L. quihoui* had higher Ka/Ks ratios (Fig. 8). Positive selection analyses of 78 single-copy protein-coding sequence genes from the ten *Ligustrum* species revealed four protein-coding genes (*accD*, c*lpP*, *ycf1*, and *ycf2*) subject to significant positive selection (*P* < 0.05). Bayes empirical Bayes (BEB) analysis revealed significant posterior probabilities for the *accD* and *rpl20* genes, with 49 positive selection sites for *accD* and four for *rpl20* (Supplementary Table 2).

### Phylogenetic results

**Figure 9:**
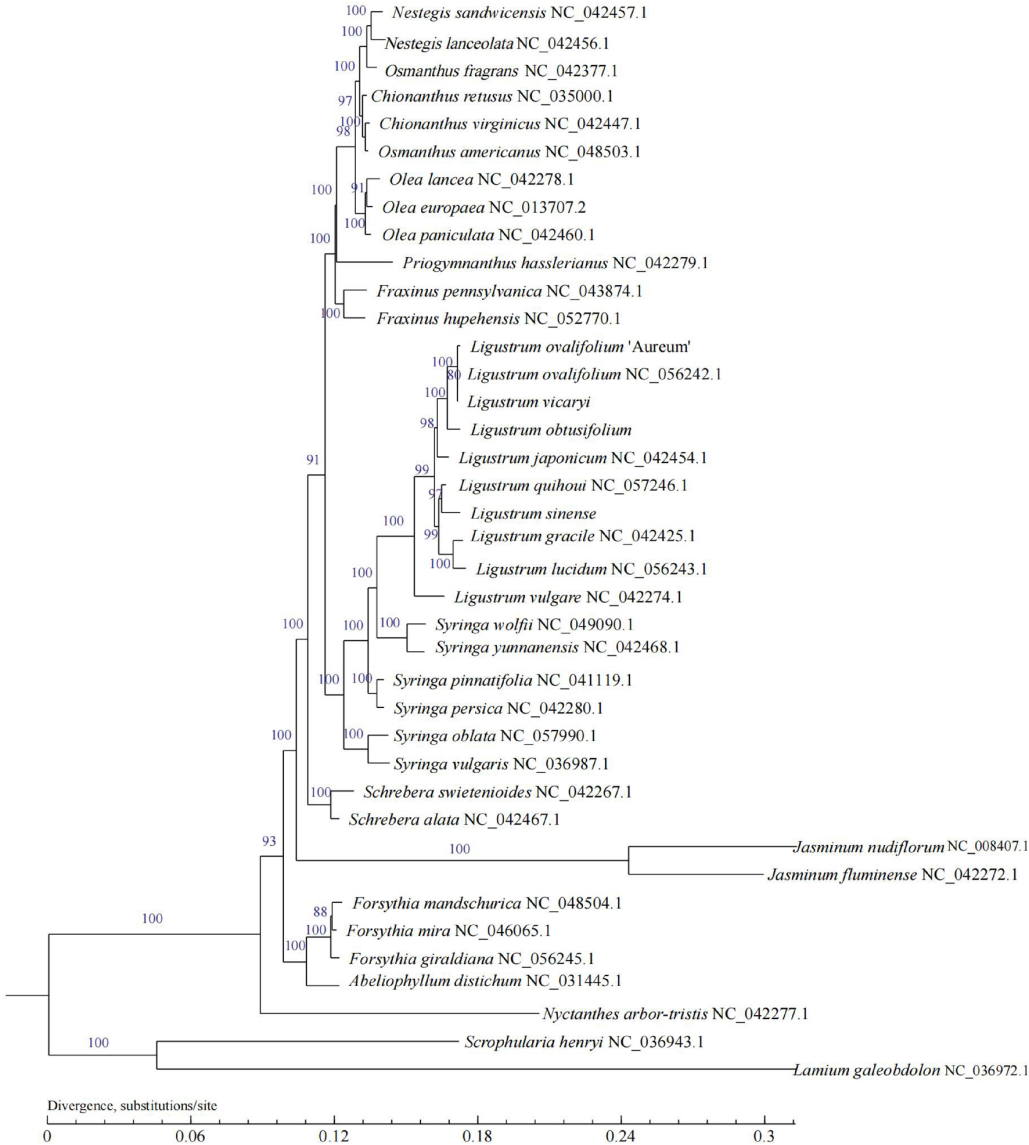
ML phylogeny of 39 species including 37 Oleaceae species and outgroups (*Scrophularia henryi* and *Lamium galeobdolon*) based on the whole chloroplast genomes.

We applied a maximum likelihood (ML) model to construct a phylogenetic tree of 37 species belonging to 13 genera in Oleaceae. The relationships among the genera in this family were well handled, and the 13 genera clustered into one branch with high support for each node, which was consistent with the botanical classification (Fig. 9). *Ligustrum* species clustered into a single monophyletic clade, with high support. The European species *L. vulgare* was the first to differentiate. *Ligustrum vicaryi*, *L. ovalifolium* ‘Aureum’, and *L. ovalifolium* formed one branch, and *L. obtusifolium* formed another. *Ligustrum* s*inense* and *L. quihoui* clustered together, and *Ligustrum* and *Syringa* were more closely related than other genera in Oleaceae.

## Discussion

### Evolution of the chloroplast genome of *Ligustrum* species

The development of rapid, low-cost high-throughput sequencing has accelerated the determination of plant cp genome sequences^17,18^. In this study, we examined the cp genomes of four *Ligustrum* species and compared their characteristics with those of six other *Ligustrum* species. Like other angiosperms, *Ligustrum* cp genomes had four zones. The cp genomes ranged from 162,185 to 166,800 bp, with a difference in size of 4,615 bp. Large variation in LSC length (2,131 bp) accounted for most of this genome-wide variation; therefore, variation in genome length appears to be mainly caused by variation in LSC length. The GC content of the ten *Ligustrum* species ranged from 37.93 to 38.06%, within the normal range of GC content in seed plant cp genomes (34–40%). The highest and lowest GC contents were in the IR (41.16–41.40%) and SSC (32.68–32.81%) regions, respectively, mainly because the IR region contains 35–39 tRNA genes and eight rRNA genes, respectively, with high GC content; high GC content in the IR region may drive its stability compared to the LSC and SSC regions. Excluding duplicate genes, a total of 114 genes were annotated to the cp genomes of the ten *Ligustrum* species, containing 82 protein-coding genes, 4 rRNA genes, and 28 tRNA genes. Among these, the *accD* gene contained one intron in *L. obtusifolium* and *L. vicaryi* but no introns in the other *Ligustrum* species; similarly, the *trnV* gene contained one intron in *L. sinense*, *L. obtusifolium*, *L. vicaryi*, and *L. ovalifolium* ‘Aureum’ but no introns in the other *Ligustrum* species. We assume that the loss of introns in *Ligustrum* species occurred during the evolutionary process. To some extent, intron loss reflects the rate of species evolution, with faster-evolving species retaining fewer ancestral introns^19,20^. Thus, plant evolution can be detected through the loss of intron polymorphisms and introns of the same gene within a species.

### Candidate DNA barcoding of genus *Ligustrum*

DNA barcoding technology has a wide range of applications in the fields of species identification, resource conservation, phylogeny, and evolution^21,22^. The cp genomes of *Ligustrum* species are generally consistent in overall gene content and arrangement. However, comparative genome analysis using mVISTA revealed relatively conserved sequences among *Ligustrum* species. Compared to the LSC and SSC regions, the sequence divergence of the IR regions was slower and the comparative conservation was due to the replication correction caused by higher gene conversion between the sequences of the two IR regions^23^. Single-copy regions have higher nucleotide diversity than IR regions, and non-coding regions have higher nucleotide diversity than coding regions, which is consistent with results from other taxa^24^. Nucleotide diversity analysis identified six highly variable regions, which were mainly located in non-coding regions. The highly variable *accD* gene sequence identified in this study was also previously identified as the most highly variable hotspot region in *Quercus*^25^, and the *ycf1* gene was also reported as a highly variable hotspot region in Papaveraceae^26^; therefore, the highly variable hotspots regions identified in this study have potential as candidate markers or DNA barcodes for inferring the phylogeny of *Ligustrum*. Further analysis of the six hotspot regions showed that the *ycf1* gene region and the *clpP1* exon region not only have high sequence variability, but also are coding region sequences, which can be accurately corrected by triplet codons. Therefore, it is more recommended that the *ycf1* gene region and the *clpP1* exon region be used as potential DNA barcodes for the identification and phylogeny of the *Ligustrum.* Jin et al. has also been found that *ycf1a* and *ycf1b* were two specific DNA barcodes of Ligustrum^17^. In this study we found that besides *ycf1* gene, the *clpP1* exon region can also be used as the candidate DNA barcode for *Ligustrum*.

### Phylogenetic tree

Using the cp genome data obtained in this study and those published for four additional species, we constructed a phylogenetic tree based on the whole cp genomes for 13 genera and 37 species of Oleaceae. Species of *Ligustrum* and *Syringa* have highly similar morphology, which can affect the discovery and identification of their fossils^27^. It is of great significance to study the relationship and taxonomic status between *Ligustrum* and *Syringa*. Based on internal and external transcribed spacer results on the rDNA of *Syringa*^28^, *Ligustrum* may have originated from *Syringa* according to *rps1* and *trnL-F* sequence analysis, such that *Syringa* is a syntaxon^29^. There is also study based on cp genomes showed that *Ligustrum* is a monophyletic group through phylogenetic analysis, while *Syringa* is a paraphyletic group, and *Ligustrum* shows the characteristics of a suspected subclass of *Syringa*^30^. In this study, an ML phylogenetic tree was constructed using the whole cp genomes. The phylogenetic results showed that *Ligustrum* and *Syringa* were clustered together and closely related. The result supports the view that *Ligustrum* is a monophyletic group, and *Syringa* is a syntaxic group, and that *Ligustrum* may originated from *Syringa*. However, compared with more than 50 species of *Ligustrum*^1^ and nearly 30 species of *Syringa*^31^, relatively few species have been subjected to complete cp genome sequencing. Therefore, the relationship and taxonomic status between *Ligustrum* and *Syringa* requires redefinition and further investigation using more genomic data.

## Materials and methods

### Plant materials and sequencing

Branches of *L. sinense*, *L. obtusifolium*, *L. vicaryi*, and *L. ovalifolium* ‘Aureum’ were collected in January 2022 at Hebei Normal University of Science and Technology. After 2 weeks of hydroponic incubation in the greenhouse of Hebei Agricultural University, the leaves were collected and stored in liquid nitrogen and sent to Shanghai Ling'en Biotechnology Co., Ltd. (Shanghai, China) for cp genome sequencing. The complete cp genome sequences of six published *Ligustrum* species were obtained from the National Center for Biotechnology Information (NCBI): *L. gracile*, *L. quihoui*, *L. japonicum*, *L. lucidum*, *L. ovalifolium*, and *L. vulgare*. *Scrophularia henryi* and *Lamium galeobdolon* were used as outgroups and the complete cp genome sequences were re-annotated for structural comparison and phylogenomic analysis using the GeSeq software^32^.

### Genome assembly and annotation

Total DNA was extracted from leaves using a plant DNA extraction kit, and the quality, integrity, and concentration of DNA were determined by agarose gel electrophoresis and spectrophotometry. To obtain high-quality clean reads, quality control of the raw reads data obtained from sequencing was performed using the Trimmomatic v0.39 software^33^ to remove low-quality sequences and junctions. Chloroplast genome assembly was performed using the NOVOPlasty v4.3 software (https://github.com/ndierckx/NOVOPlasty)^34^. Sequences with sufficiently high coverage depth and long assembly length were selected as candidate sequences, and cp scaffolds were confirmed by comparison with the NT library and overlapped sequences. Validated the assembly results by mapping reads to the assembled sequence and show the results in the Supplemental Figure, and the specific depth results has be placed in the Supplemental file-Depths. BLAST searching^35^ was performed to compare the assembled sequences with cp reference genome sequences of the proximal species (*L.quihoui*, NC_057246.1) to determine the initial position and orientation of the cp assembly sequence and determine the possible cp partitioning structure (LSC/IR/SSC) to obtain the final cp genome sequence. The GeSeq software^32^ was used to predict the cp genome for coding proteins, tRNA, and rRNA genes, and then the predicted initial genes were made de-redundant and the first and last genes and exon/intron boundaries were manually corrected to obtain a highly accurate gene set. Finally, the Chloroplot software (https://irscope.shinyapps.io/Chloroplot/)^36^ was used to generate a physical map of the fully annotated cp genome.

### Codon usage indices

The CodonW v1.4.4 program^37^ was used to evaluate gene codon usage in terms of five indices: the CAI, CBI, FOP, GC3s, and ENc.

### SSRs and repeat sequence analysis

SSRs in the cp genomes of ten *Ligustrum* species were analyzed using the MISA software^38^, with the parameters 1–8, 2–5, 3–4, 4–3, 5–3, and 6–3, such that there were no fewer than eight mononucleotide repeats, no fewer than five dinucleotide repeats, and no fewer than four trinucleotide repeats, and there were at least three tetranucleotide, pentanucleotide, and hexanucleotides repeats. The REPuter software^39^ was used to identify forward (F), reverse (R), palindrome (P), and complementary (C) repeats in *Ligustrum* species that met the requirements of a minimum repeat size of 30 bp and 90% or greater sequence identity (Hamming distance = 3). Tandem repeats were detected using the Tandem Repeats Finder v4.04 software^40^, with the default parameters.

### Comparative analysis of chloroplast genomes

The mVISTA program in the shuffle-Lagan model^41^ was used to compare the cp genome sequences of the ten *Ligustrum* species using the *L. sinense* cp genome as a reference. The DnaSP v5.10 software^42^ was used to calculate the Pi values of the LSC, SSC, and IR regions among the ten *Ligustrum* species, and to identify divergence hotspot regions within the genome for evolutionary analysis. The step size was set to 200 bp and the window length to 300 bp. The IRscope software (https://irscope.shinyapps.io/irapp/)^43^ was used to draw an IR boundary map and compare IR boundary characteristics among the cp genomes of *Ligustrum* species.

### Ka/Ks and forward selection analysis

To analyze the effect of environmental stress on the evolution of *Ligustrum*, we calculated the Ka/Ks ratio for all species. The Muscle software was used to compare gene sequences, and then the KaKs_Calculator2 software^44^ was used to calculate Ka and Ks values, using the default parameters, except for -c 11-m MS. The optimized branch-site model^45^ and BEB^46^ methods were used to identify genes of the *Ligustrum* species subjected to positive selection. The TrimAl v1.4 software^47^ was used to trim the multi-sequence alignment results of single-gene nucleic acids; then, the codeml program in the PAML v4.9 package was used for branch-site model analysis. We calculated the null hypothesis (null model, model = 2, NSsites = 2, fix-omega = 1, omega = 1) and alternative hypothesis (alternative model, model = 2, NSsites = 2, fix-omega = 0, Omega = 0.2). We ran a Chi-square test in PAML for the likelihood ratio test^48^, where positively selected genes were evaluated at a level of *P* < 0.05. Finally, the BEB method was used to calculate the posterior probabilities of amino acid sites to determine whether the sites were positively selected.

### Phylogenomic analysis

Complete cp genome sequences of Oleaceae, particularly *Ligustrum*, were selected from the NCBI database, and phylogenetic analysis was performed using the ten *Ligustrum* species examined in this study, and 27 other Oleaceae species. *S. henryi* (NC_036943.1) and *L. galeobdolon* (NC_036972.1) were selected as outgroups. The complete cp genome sequences were used for tree construction. They were extracted and aligned using MAFFT v7.458^49^, and the alignment was trimmed by Gblocks_0.91b^50^ to remove low-quality regions with the parameters: -t = d -b4 = 5 -b5 = h. ML phylogenetic tree based on the best-fit model of GTR + I + G was conducted using PhyML 3.0 (http://www.atgc-montpellier.fr//phyml/)^51^. The Best-fit model by jModelTest 2.1.10^52^, according to Bayesian information criterion (BIC) and the robustness of the topology was estimated using 1000 bootstrap replicates.

## Conclusion

In this study, the cp genomes of four *Ligustrum* species were assembled and annotated, and a series of characteristic analyses were performed using six additional published *Ligustrum* species. The results showed that the cp genome of *Ligustrum* species has a tetrad structure, with similar and conserved genome structures and gene numbers. The total length of the cp genome was 162,185–166,800 bp, and the GC content ranged from 37.93 to 38.06%. Six hotspot regions were identified from multiple sequence alignment of *Ligustrum*; the *ycf1* gene region and the *clpP1* exon region can be used as potential DNA barcodes for the identification and phylogeny of the genus *Ligustrum*. The identification of four positive-selection genes in this study will contribute to our understanding of the adaptation of *Ligustrum* species to the environment. Based on the whole cp genomes, we constructed an evolutionary tree of Oleaceae species, which showed that 13 genera in Oleaceae were clustered into one branch, each node having a high support rate, and *Ligustrum* and *Syringa* were the most closely related groups. Through sequencing and analysis of the cp genomes of *Ligustrum* species, the results of this study provide a basis for identifying and elucidating the phylogenetic relationships of *Ligustrum* species.

### Specimen collection

The plant material was collected with the owner's permission and in accordance with relevant guidelines and regulations.

## Supplementary Information

Supplementary Information 1.

Supplementary Information 2.

Supplementary Information 3.

Supplementary Information 4.

Supplementary Information 5.

Supplementary Information 6.

## Data Availability

The original contributions provided in the study are publicly available and can be found at NCBI (SRR21590286, SRR21590285, SRR21590284, SRR21590283).
