# Supplementary material for: Complete chloroplast genomes and comparative analysis of Ligustrum species
Source: Sci Rep. 2023 Jan 5;13:212. doi: 10.1038/s41598-022-26884-7 (PMC9814286; doi:10.1038/s41598-022-26884-7)
Supplement: Supplementary file 1 — Supplementary Information 1. [file 41598_2022_26884_MOESM1_ESM.docx]

**Supplementary Materials**

Supplementary materials can be found at Table S1. Intron length (bp) of chlorplast genes of ten *Ligustrum* species; Table S2. Positive-selection analysis of single copy genes of ten *Ligustrum* species; Supplemental file-SNP; Supplemental Figure and Supplemental file-Depths.
