# Supplementary material for: Complete chloroplast genomes and comparative analysis of Ligustrum species
Source: Sci Rep. 2023 Jan 5;13:212. doi: 10.1038/s41598-022-26884-7 (PMC9814286; doi:10.1038/s41598-022-26884-7)
Supplement: Supplementary file 4 — Supplementary Information 4. [file 41598_2022_26884_MOESM4_ESM.pdf]

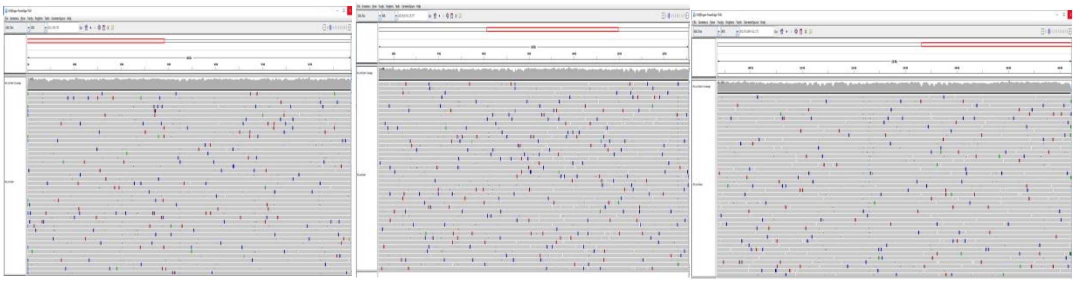

Supplemental Figure 1. The IGV screen capture of *Ligustrum sinense*

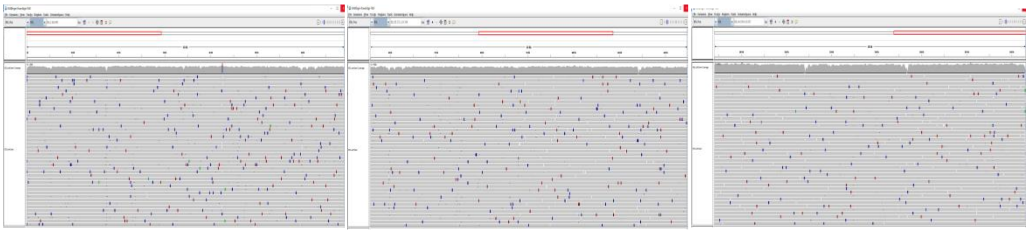

Supplemental Figure 2. The IGV screen capture of *Ligustrum obtusifolium*

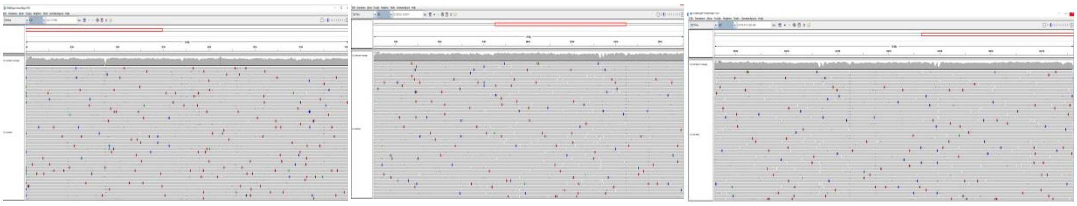

Supplemental Figure 3. The IGV screen capture of *Ligustrum vicaryi*

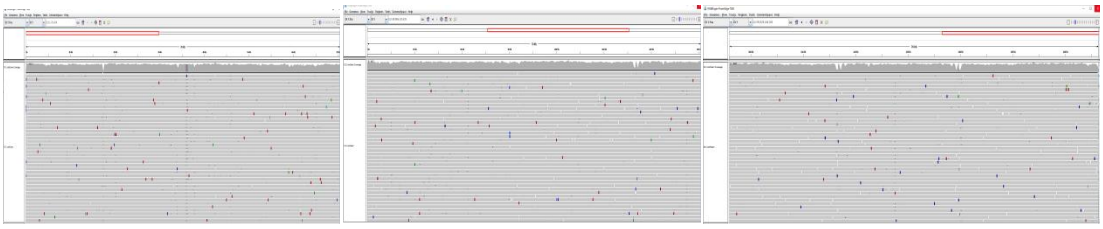

Supplemental Figure 4. The IGV screen capture of *Ligustrum ovalifolium* 'Aureum'
